# Mass medicine vs. personalized medicine: from mathematical methods to regulatory implications

**DOI:** 10.3389/fphys.2025.1649114

**Published:** 2025-07-14

**Authors:** Inderpal S. Randhawa, Grigori Sigalov

**Affiliations:** Food Allergy Institute, Long Beach, CA, United States

**Keywords:** AI, machine learning, adaptive clinical trials, patient variability, subgroup analysis, multivariable models, predictive modeling, precision medicine

## Abstract

Clinical trials of a treatment in traditional mass medicine are based on the concept of proof of efficacy. It must be proven for a group of subjects that meet certain selection criteria. Subject variability must be demonstrated to exist and yet not to invalidate the proof of efficacy. If so, it is assumed that new patients meeting the same selection criteria would have a uniform response to treatment, irrespective of their individual traits. However, the variability that can be ignored for a group should not be ignored for an individual. Standard statistical methods are designed to estimate an average effect size for large enough groups, but they cannot predict an expected effect size for a single patient. Such predictions based on the patient’s individual characteristics, rather than on their classification as a member of a target population or study group, are possible in personalized medicine. The latter employs multivariable predictive models via advanced mathematical methods implemented in Artificial Intelligence (AI), and it incorporates the subject variability in the predictive models to improve their accuracy and selectivity. There is a common misconception that personalized medicine belongs in a narrow area of rare diseases or genotype-guided care. In this paper, we argue that AI has potential to improve the treatment success estimates in traditional mass medicine as well at no extra cost to researchers. The clinical trial data on subject variability that are already routinely collected only need to be analyzed and interpreted using the methods of personalized medicine. To implement such improvements in medical practice, they need to be acknowledged and regulated by FDA and its counterparts in other countries.

## 1 Introduction

Historically, medicine has aimed to treat large groups of people with similar symptoms using the same intervention (e.g., drug, operation, treatment). This approach, referred to as mass medicine, has proven effective in the prevention and treatment of widespread diseases such as smallpox, tuberculosis, syphilis, and polio. To test a new drug, typically, a placebo-controlled clinical trial is performed with a few hundred participants [Walsh et al. (2014) report a median sample size of 682 patients based on meta-analysis of 399 trials]. Treatment success is often measured by comparing the percentage of positive outcomes in the intervention group against the control group. The assumption is that future patients meeting the same selection criteria as trial participants will exhibit a similar rate of success or effect size of treatment.

The group of subjects selected for a trial must constitute a representative sample of the target population. Variation of patient characteristics within the study group must be shown to be similar to the respective distribution within the target population, though no exact metrics of similarity are specified by the regulators such as FDA. The trial data analysis must prove the treatment’s efficacy despite the patient variability. This is often done by using Student’s t-test to show the statistical significance of the differences between the intervention group and the placebo control group. Once the patient variability is demonstrated to be irrelevant to the treatment efficacy, it is also ignored when it comes to the estimate of the treatment’s success rate or effect size. This approach amounts to a zero-parameter model for all patients that belong to the final analyzed population. Thus, to evaluate expected chances of success for new patients of mass medicine, medical professionals rely on simple statistical data (e.g., the average effect size and its standard deviation or confidence interval [CI]) that are the same for all patients that meet the treatment criteria, and on empirical experience, rather than on explicit predictive modeling, with rare exceptions.

Over recent years, we witnessed considerable progress in the application of Machine Learning (ML) and, more generally, Artificial Intelligence (AI) in personalized medicine, which is often employed to deal with rare diseases, atypical patient conditions, and genotype-based healthcare. In personalized medicine treatment is tailored for the individual patient rather than a target population, which might be too small for meaningful statistical analysis. Personalized medicine aims at maximizing the effect size for a narrow sample of subjects or even a single patient, in contrast to the goal of maximizing the target population coverage with the minimum demonstrably non-random effect size in mass medicine.

The novel AI approaches developed for personalized medicine had no visible effect on traditional mass medicine, which is widely considered to be an established field where classical statistical methods are sufficient for clinical trial data analysis and interpretation. In this paper, we would like to challenge this view. While the account for patient variability via multivariable models is as much at the methodological foundation of personalized medicine as the lack thereof is one of the governing principles of mass medicine, the gap between them is not as formidable as it is often perceived, and it could (and should) be bridged, for the benefit of both medical paradigms and for their patients.

The natural pathway to such a synthesis of ideas would be to proactively employ the mathematical apparatus of personalized medicine, that is, multivariable modeling implemented in the form of appropriate AI methods, for analysis and interpretation of data obtained in clinical trials of standard designs used in mass medicine. In what follows, we plan to demonstrate that enough useful data are already being collected in such trials but not utilized to the full extent. Therefore, the advances in predicting the treatment outcomes for new patients could come at zero additional cost of data acquisition, while the extra costs of data maintenance, storage, analysis, etc., are comparably low.

Below, we examine the key distinctions between traditional statistical tools used in mass medicine and advanced AI models employed in personalized medicine. We provide examples of published clinical trial cases and discuss how some data, collected but not fully analyzed, could be used to better explain the outcomes. We also suggest that the mathematical differences between the methods of classical statistics and modern AI should be reflected in evolving regulatory requirements to accommodate novel clinical trial designs and approaches to data analysis.

## 2 Statistical measures of treatment efficacy in mass medicine and personalized medicine

For the benefit of readers coming from different backgrounds, we will provide a correspondence between the terms used to describe the logic and data flow of clinical trials in medical literature, on one hand, and in mathematics, on the other hand. Here, we use the term ‘mathematics’ in a wide sense so as to include its applied fields such as statistics, ML, and AI.

The concept behind the search for a new treatment, i.e., a drug, through a clinical trial is very simple: if a large group of patients is selected from the target population using a fixed set of selection criteria, and the drug is tested successfully on members of that group, then one can expect similar results when the same drug is administered to other subjects from the target population. Typical general design of a placebo-controlled clinical trial is as follows: the test group is randomly split into the intervention and the control groups, the former receiving and the latter not receiving the drug. If there is a statistically significant difference between the occurrence of the desired positive outcome in the intervention group as compared to the control group, then it must be due to the effect of the drug rather than pure chance.

In mathematical terms, one selects a class of objects that satisfy the same conditions and are therefore assumed to have the same statistical property with respect to a certain test (corresponding to the medical intervention). When the test is applied to an object, the latter either passes the test (positive outcome) or fails it. One cannot know in advance whether any particular object would pass or fail the test, but it is assumed that the probability of passing is the same for any randomly selected subset of the class, within a confidence interval depending on the subset size. This assumption makes it possible to estimate the probability of passing the test for a selected subset (subjects of a clinical trial), and then use it to predict the average chances of passing for the rest of the class (new patients). In the terms of AI, a model is trained and validated on a training dataset collected during the clinical trial. Finally, the trained model is used to predict the probability of the positive outcome in out-of-sample cases, that is, for new patients.

A model in which all objects are assumed to have the same probability of passing the test is parameter-free and therefore the easiest to design and test. It may not be the most accurate though for test objects that are not identical, such as humans—as opposed to coins flipped or dice thrown in the classical experiments at the foundation of probability theory and statistical science.

### 2.1 Account of patient variability in clinical trials via subgroup analysis

Clinical trial participants are not identical but are expected to exhibit similar treatment responses, at least statistically. To prove that different individuals could show a similar response, one must demonstrate that their differences would not affect the trial’s conclusions. This is done, in particular, via subgroup analysis. For example, in a study of the effect of feeding newborn infants with a Cow Milk Formula (CMF) on the onset of Cow Milk Allergy by their second birthday (Urashima et al., 2019) the subgroup selection was based on tertiles of serum 25(OH)D levels, a biomarker of vitamin D status. The intervention, defined here by avoiding CMF for a period of time after birth, was found to be significant (P = 0.02) for the middle tertile, but not for the other two. The study lists 16 additional participant characteristics (APCs) such as parental age and the incidence of several allergies in the infants’ parents and siblings. The mean numerical values for these characteristics are provided only to claim—without an analysis of statistical significance or correlation—that they are similar across the intervention and control groups. Thus, it is demonstrated that there is a certain variation of APCs among the trial subjects, and yet, the intervention was effective for the middle tertile of the intervention group. Therefore, a conclusion is made that those APCs are not essential and could be ignored when selecting new patients to whom the intervention in question could be recommended.

While this is a valid and prevalent train of thought, it could be that including the additional data—already at the authors’ disposal—on 16 additional parameters in the numerical analysis would provide additional insight, compared to ignoring those data summarily. For example, they could be used for subgroup analysis. It is not clear why CMF avoidance was effective in the middle tertile (21–36 ng/mL) but not the lower or the upper ones. Could it be that some of the 16 known APCs had something to do with this result? Is it possible that the subgroup for which the intervention was effective would include a larger percentage of the participants if the subgroup split was done differently? Such questions were neither answered nor asked in Urashima et al. (2019). We will discuss below why not, in our opinion, and how a clinical trial could benefit if they were.

In a study of the effect of peanut avoidance on peanut allergy in young children (Du Toit et al., 2015), two subgroups were defined by the results of a skin-prick test (SPT) (no wheel or 1–4 mm wheel) at the beginning of the trial. In addition, 8 APCs were recorded. Among them, the results of the trial grouped by race showed that the effect of peanut avoidance was statistically significant for all races but one. The race subgroup that was an exception only had 8 participants, compared to 24 to 460 in other race subgroups. The primary outcome was defined as the proportion of participants with peanut allergy at 60 months of age. This proportion turned out to be consistent yet somewhat different across the race subgroups. While it is clear that the final conclusions of the study are valid for all races (possibly barring one for which there was not enough data), it would be interesting to see if race had any effect on the numerical value of peanut allergy occurrence. Averaged data for the remaining 7 additional participant characteristics were provided but not used for analysis or discussion. A more comprehensive analysis could have identified predictors of treatment effectiveness.

In mathematical terms, subgroup analysis introduces a discrete model parameter for each subgroup criterion. In Urashima et al. (2019), it was the tertile ordinary number (1, 2, or 3) depending on serum 25(OH)D level, a single parameter (covariate). Bradshaw et al. (2023) performed exploratory subgroup analysis according to filaggrin (FLG) genotype in a study of emollient application’s effect on preventing atopic dermatitis and other allergies. There were 3 subgroups (one, two, or no mutations) for a single parameter, the FLG genotype. In Du Toit et al. (2015), one parameter was the SPT result (negative or positive, i.e., a Boolean value) and the other was race (a categorical variable with 5 possible values). In these studies, it would be possible to create a 1- or 2-parameter model, respectively, which would be a step up from a parameter-free model in the sense of the model’s potential predictive power. In the research quoted above, the covariates were used to analyze the treatment effectiveness across different parameter values. But they could also be used to predict the probability of the primary outcome (PPO) depending on those parameters, if such correlation turned out to exist. Moreover, the APCs that were numerical by nature, such as the serum 25(OH)D levels, did not have to be necessarily grouped into tertiles or other range-based subgroups. There is a chance that PPO depended on the numerical value of that parameter directly, not just on the subgroup it falls into. Finally, all the APCs that were recorded but not used in the analysis could become predictors in an ML model. Pending numerical analysis, some of them might turn out to be significant predictors of PPO.

### 2.2 Subgroup analysis in multiple dimensions: parameter space fragmentation issue

FDA guidelines (Guidance for Industry, 1998) recommend diverse clinical trial populations to mirror real-world demographics. Usually, the APCs, also referred to as Demographics and Baseline Characteristics, are reported to demonstrate such diversity and, therefore, a lack of selection bias. Each APC potentially could be used as a parameter to stratify the study cohorts for further subgroup analysis. We will call them subgroup stratification parameters (SSPs). Mathematically, each of them corresponds to an additional axis on a graph in a visual presentation of data (at least when the number of SSPs is 3 or fewer), or to an additional degree of freedom (DoF) of a possible ML model. Often, multiple APCs are listed yet only one of them is used as an SSP in subgroup analysis (Urashima et al., 2019; Du Toit et al., 2015).

If the entire group of study subjects is varied enough in respect to each SSP (i.e., along each DoF) so as to be representative of the target population, could the same be claimed for each subgroup? The short answer is, not necessarily. That would only be true if the subgroup counts do not have a strong correlation across SSPs, if more than one of them is present. To illustrate this statement, consider a hypothetical clinical trial with 1,000 subjects stratified into subgroups by sex (male or female) and age (under 18 and 18 or above). Suppose the number of subjects in each cross-stratified subgroup are as given in Table 1.

**TABLE 1:** Cross-stratification by age and sex of participants in a hypothetical clinical trial.

Age/Sex	Male	Female	Total by age
Under 18	210	18	228 (22.8%)
18 and above	275	497	772 (77.2%)
Total by sex	485 (48.5%)	515 (51.5%)	

The overall percentage of female subjects (51.5%) and the overall percentage of subjects aged under 18 (22.8%) closely mirror the US population. However, females are grossly underrepresented in the cohort of subjects under 18 (18 of 228, or 7.9%). Also, subjects under 18 are grossly underrepresented in the female cohort (18 of 515, or 3.5%). This observation of a strong correlation could be verified by a chi-square test (in this case, χ^2^ = 224.03, which is much greater than the critical value of 3.84) or another statistical metric appropriate for the data (Howell, 2013). If the purpose of subgroup analysis was to “explore the uniformity of any treatment effects found overall” (Guidance for Industry, 1998) and to verify that the conclusions of the trial for the entire group of 1,000 subjects were valid across different demographic groups (Statistical Guidance on Reporting Results, 2007), then it would not be satisfactory to only perform the subgroup analysis for age alone and sex alone. Due to a strong correlation between these parameters in the subject count, the subgroup analysis should then be done for each of the 4 cross-parameter subgroups (male children, female children, male adults, female adults).

This would create a problem, however, because a subgroup of 18 female children may be too small for meaningful statistical analysis. In general, with each additional SSP and each additional level (stratum) in the respective stratification, the selected trial population becomes further fragmented. For example, in a study of efficacy of Tezepelumab in asthma, Corren et al. (2023) use biomarker subgroups based on FEIA (2 levels), ICS dose (2 levels), FENO (4 levels), BEC (4 levels). Also, they report APCs (which are not but could be used as SSPs) such as sex, allergy status, number of exacerbations, history of nasal polyps, OCS use at entry (2 levels each) and BMI (3 levels). If all of these identifiers were used to create cross-parameter subgroups, then the parameter space of the design would be fragmented into 2^7^ × 3 × 4^2^ = 6,144 subgroups. Given a total of 665 subjects in the intervention group, this would mean that most of the 6,144 subgroups would be assigned too few study subjects (or none at all) for statistical analysis. This does not mean, however, that such low-populated subgroups do not exist in the target population. Vital et al. (2022) utilize 8 two-level subgroups as well as stratification based on race (4 levels) and region (5 levels), for a total of 2^8^ × 4 × 5 = 5,120 potential cross-parameter subgroups for a study population of 360 subjects in the intervention group. Necessarily, subgroup analysis is performed for each subgroup taken alone, but not for cross-parameter combinations of them. As shown above, this might lead to misinterpretation of the trial’s results due to interactions between subgroups.

### 2.3 Broader inclusion criteria mean smaller average effect size

The study population in any clinical trial is limited by practical considerations of subject availability, patient consent, data acquisition, and overall per-subject costs. It would not be reasonable nor possible to obtain enough subjects to populate each and every cross-parameter subgroup for sound statistical analysis. As recommended by FDA (Guidance for Industry, 1998), the present covariates (APCs) are reported to show the study population diversity or used as SSPs to define subgroups for exploratory subgroup analysis. Once it has been established that the population’s variation with respect to these covariates does not compromise the overall uniformity of any treatment effects, the APCs are no longer considered, and the trial endpoints (usually, the treatment efficacy and safety) are normally not adjusted for any APC variation.

This approach stems from the goal of mass medicine to treat as high a proportion of the target population as possible using the same intervention. When the treatment effect can be measured numerically, the widest coverage of the target population is achieved when the treatment’s metric is permitted to have the lowest value (assuming that a higher metric corresponds to a stronger positive effect) above a certain threshold. For example, Chan et al. (2020) use SCORAD and a Life Quality Index, while Maurer et al. (2013) use an itch-severity score as numerical metrics of treatment effect. Typically, Student’s t-test is used to verify the statistical significance of the difference between the intervention and placebo groups. When the difference is just enough to reject the null hypothesis (e.g., with P value of 0.05 as a threshold), the t-test proves the treatment efficacy irrespective of its effect size (e.g., the numerical difference between the pre- and post-treatment metrics). A greater average effect size could be achieved if a lower P value is chosen as a threshold, but this would come at the cost of fewer trial subjects meeting the stronger selection criteria. And vice versa, broader uniform coverage of the target population may reduce the average treatment effect size, though it can still remain strong enough to demonstrate efficacy—i.e., statistical significance.

### 2.4 Resolution of parameter space fragmentation issue via transition from subgroup analysis to ML modeling

When the goal is to optimize the treatment effect size, albeit for a smaller group of patients or even a single patient, rather than to treat a wider population with a uniform yet lower average success rate, one enters the realm of personalized medicine. It uses the same principles to collect clinical data, but it processes them differently because of a different endpoint. Instead of proving that covariates could be ignored and that data for subjects with different APC values could be summarized to produce a variable averaged (ideally) over the entire study population, personalized medicine seeks to build a model in which every essential APC is included, and its effect on the endpoint variable is modeled to best fit the observed data distribution over the entire trial population. ML models, though not the only type of AI methods used in medicine, turn out to be especially well-suited for the task (Sinha et al., 2021; Randhawa et al., 2023; Liu et al., 2019; Randhawa and Marsteller, 2024).

The general idea behind a clinical trial for the purposes of mass medicine is that, to prove the treatment efficacy for a target population, one must prove it for a statistically representative subset (randomized sample) of that population. A trial must show that the subjects within the selected sample are similar enough, despite the APC variations, to make conclusions about the average expected outcome for the entire group. Since individual APCs of the trial subjects are discarded, one cannot predict the effectiveness of treatment for a particular individual patient. One can only predict the mean effectiveness for a population to which that patient belongs, according to the selection criteria.

On the other hand, an ML model is designed to make a prediction for an individual out-of-sample (new) patient using the values of all essential APC parameters (predictors), not just based on the fact that this patient meets the same selection criteria as the members of the clinical trial’s final analyzed population. The predictive power of an ML model is due to its utilization, at the stages of model design, training, validation, and testing, of the data set for the entire trial population, including subjects with dissimilar APC values. Outliers and inessential parameters can be automatically detected and removed. Values of continuous variables no longer need to be grouped into finite intervals for subgroup analysis but can be used as continuous predictors in a regression model. This resolves the issue of parameter space fragmentation. Possible correlations between the predictors are automatically handled when the model is trained, redundant predictors are removed, and the contributions of remaining predictors are estimated along with their P-values and CI bounds. As such, a regression ML model amounts to the classical statistics treatment of data in more than one dimension. Any additional patient parameters become a meaningful data source that helps to elucidate the effect of those APCs on the treatment outcome and predict it more accurately for each individual patient. It is worth emphasizing that the data required for such analysis are already routinely collected in traditional clinical trials (Urashima et al., 2019; Du Toit et al., 2015; Bradshaw et al., 2023; Corren et al., 2023; Vital et al., 2022; Chan et al., 2020; Maurer et al., 2013; Liu et al., 2019).

Subgroup analysis uses established statistical tools to prove that the treatment efficacy is uniform across certain subgroups and to find those subgroups where this is not the case. In other words, it establishes the selection criteria for the treatment. ML algorithms also possess proven mathematical tools to determine which study subjects should not be part of the final analyzed population (i.e., to find outliers), to select parameters that are essential for explaining the outcome variability, and to evaluate the expected prediction error (Kuhn and Johnson, 2019). Therefore, it is not only technically possible but advisable to consider all available APCs as candidate predictors for an ML model.

For example, in a study of peanut anaphylaxis risk in children and young adults (Randhawa et al., 2023), an automatic selection algorithm picked 10 essential predictors out of 243 available demographic and biochemical parameters. The resulting ML model predicted the severity of possible anaphylactic reaction to peanut for individual patients with a recall of 95.2% and area under the curve (AUROC) > 0.99.

In a study of 214 children with cow’s milk anaphylaxis (Randhawa and Marsteller, 2024), 8 variables proved to be essential (P < 0.05). It may be useful as a demonstration on how large-scale data can build ML platforms to actually drive individual outcomes toward diagnosis and remission of dairy allergy.

Paper (Sinha et al., 2021) is another example of a case study that demonstrates how ML can be utilized to uncover clinically meaningful subgroups, improve treatment targeting, and explain heterogeneity of treatment effects. This study applied unsupervised ML clustering to patient data from three randomized controlled trials on Acute Respiratory Distress Syndrome (ARDS)—FACTT, ALVEOLI, and ARMA—to identify distinct biological subphenotypes (hyperinflammatory vs. hypoinflammatory). The ML-derived subgroups showed significantly different responses to interventions—e.g., fluid management strategies had divergent effects depending on the ARDS subtype. These insights were not apparent in the original average-effect analyses.

### 2.5 Mathematical concept of probability and its clinical interpretation

Consider a medical professional weighing a certain treatment for a patient. For simplicity, let us assume that the primary outcome is qualitative (positive or negative). A proof of treatment efficacy by simple, time-proven statistical tools, based on a clinical trial of a large enough number of similar patients, tells the doctor that the treatment should have a positive effect for that particular patient with a certain published probability. But a more accurate statement should be as follows: given a large enough number of patients like one the doctor is considering, provided that all of them meet the selection criteria of the trial’s final analyzed population, the treatment should have a positive effect for a certain percentage of them.

This percentage is a fixed number that is the same for all members of the respective population, and as such, it should not be necessarily interpreted as the probability of a positive effect for an individual patient. A train of thought saying, for example, “if the treatment had positive outcome for 70% of a responder subgroup, then there is a 70% probability it would work for a new patient meeting the same criteria” exemplifies a misinterpretation of the mathematical concept of probability. The trial subjects have different values of their covariates, or APCs. Some of these APCs may correlate with the primary outcome. It is possible that the APC variations within the sample lead to a systematic bias for individual patients, but positive and negative biases of different patients cancel each other on average. If the APC data are discarded as irrelevant (which is correct in the statistical but not individual sense), and their possible correlation with the outcome is not analyzed and reported, then one cannot predict whether the APCs of a new patient would result in a bias compared to the reported average value for the group, let alone evaluate such bias. Even if the doctor knows from experience that some of the patient’s APCs, for example, age, BMI, blood panel data, etc., indicate that the patient might be more or less susceptible to treatment than average, there is no tool at the doctor’s disposal to adjust the average success expectancy numerically if those APCs are not part of a predictive model. If they are, then some of the outcome’s variability in the clinical trial can be explained by the APC variation, and that information could be derived from the group data and then used to improve the accuracy of prediction for a new patient.

Using more formal terms, a statistical model obtained by testing a group of objects and retaining only the average value of the endpoint variable and its standard deviation (SD) is parameter-free. Classical statistical science deals with objects such as coins or dice, and it is (correctly) assumed that all objects are practically identical. Therefore, when tested, they exhibit different outcomes randomly, without a bias. Subjects of medical trials are never identical; on the contrary, it is required that the trial population has a distribution of APCs that is representative of the target population. However, since the outcomes are averaged over all subjects, any existing bias due to APC variability cancels out. This creates a parameter-free model that is valid for a group with the same distribution of APCs, but that may not be accurate for a subgroup with a narrow distribution of APCs (e.g., females of the same age), or for a single patient. If the APCs do not correlate with the outcomes of the trial, then the outcome variability is truly random, and the parameter-free model is correct. Note that this would not be a typical situation, and then the absence of correlation should be proven statistically, just as the statistical significance of efficacy is routinely proven. However, if such a correlation exists but it is ignored, then a systematic bias is, in effect, misinterpreted as random variation. In that case, not only a single patient but also any subgroup with different distribution of APCs may demonstrate an uncompensated bias.

As a simple example, imagine a large set of asymmetric dice with a broad distribution of randomly distorted shapes. If you throw all dice together and count the outcomes 1 to 6, you may find that they have equal probabilities, just like in a set of perfectly shaped dice. However, every single asymmetric die, when thrown alone, would show a systematic bias toward some outcomes. This behavior is only hidden in a group with a specific distribution of shapes. A group-based frequency of an outcome can only be interpreted as the probability of a random event if all objects in the group are identical.

### 2.6 Clinical trial data interpretation via parameter-free vs. multivariable models

Consider a hypothetical clinical study that recorded forced expiratory volume in one second (FEV_1_) in 500 asthma patients aged 25–75. Suppose that the only recorded APC was age, and that the efficacy is demonstrated for all age groups. We will compare possible reports of the study results analyzed within two paradigms discussed above. The data were randomly generated for the purpose of this demonstration to be normally distributed around the mean, which is a linear function of age.

**FIGURE 1:**
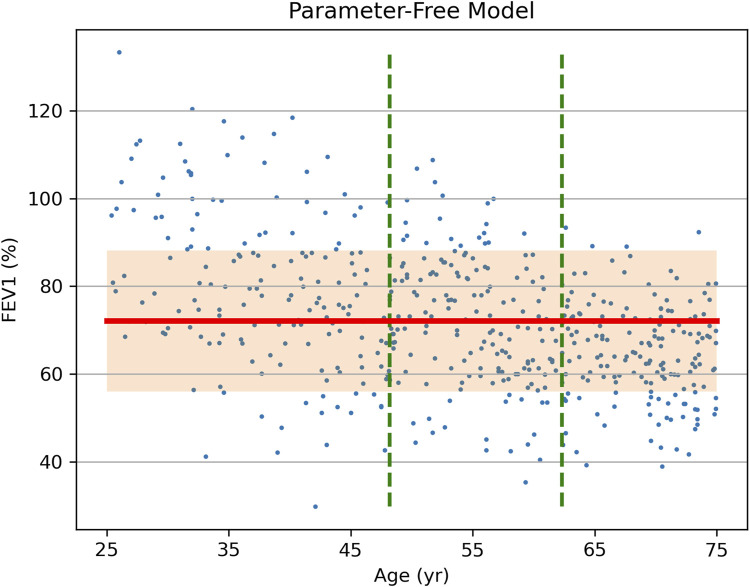
Source data (FEV_1_ vs. Age, dots) of a hypothetical clinical study of treatment effect on FEV_1_ in asthma patients and the results of data analysis via a parameter-free statistical model. Vertical dashed lines show stratification into tertiles by age. The overall study population-mean FEV_1_ is shown as a bold horizontal line, with the shaded area representing one standard deviation (SD = 16.1) above and below it.

Using the traditional approach, the participants are divided into 3 tertile subgroups stratified by age, with the mean FEV_1_ and standard deviation (SD) shown in Table 2. Figure 1 presents the source data points, the position of the overall mean FEV_1_, and the band that is offset from the mean by 1 SD in each direction. Clearly, the data suggest that a typical FEV_1_ decreases with age, but a parameter-free model has no means of incorporating this interaction, even if it is discovered and noted. As a result, the overall mean FEV_1_ better represents the results for the middle tertile than the lower and upper tertiles. This leads to the overall SD (16.1) being greater than the SD for the middle and upper tertiles (14.4 and 11.0, respectively).

**TABLE 2:** Participant Characteristics in a hypothetical clinical study of treatment effect on FEV_1_ in asthma patients.

Subgroup	Age (yr)	FEV_1_ (SD) (%)
Overall (n = 500)	25.4–74.9	72.1 (16.1)
Subgroups stratified by age		
Lower tertile (n = 165)	25.4–48.1	79.5 (18.5)
Middle tertile (n = 166)	48.2–61.8	72.1 (14.4)
Upper tertile (n = 169)	62.3–74.9	64.8 (11.0)

**FIGURE 2:**
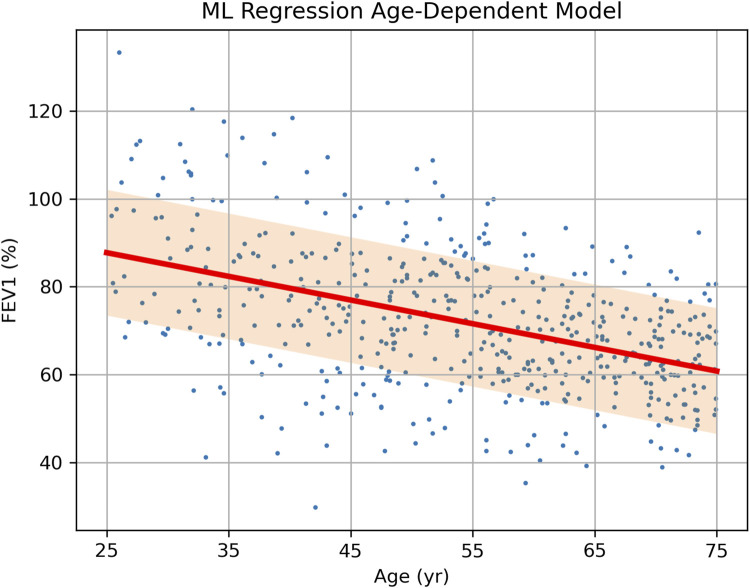
Source data (FEV_1_ vs. Age, dots) of a hypothetical clinical study of treatment effect on FEV_1_ in asthma patients and the results of data analysis via a single-parameter linear regression model. The prediction of FEV_1_ depending on age is shown as a bold sloped line, with the shaded area representing one standard deviation (SD = 14.3) above and below it.

If the same raw data are analyzed using an ML linear regression model with age as a predictor, the result is not a fixed number but a formula [FEV_1_] = A + B × [Age], where intercept A and slope B are shown in Table 3, each along with its own error and CI. Using this formula or the trained ML model, the expected FEV_1_ and its error margin can be predicted individually for a new patient using their age, as opposed to using the overall mean FEV_1_ and error obtained for all age groups. The regression has a root-mean square error (RMSE) of 14.3 (compare it to SDs in Table 2) and it approximates the true data more closely and uniformly across all ages (Figure 2).

**TABLE 3:** Parameters of the ML linear regression model trained on the source data of the hypothetical clinical study of treatment effect on FEV_1_ in asthma patients.

	coef	std err	t	P>|t|	[0.025	0.975]
Intercept	101.2049	2.646	38.245	0.000	96.006	106.404
Age	−0.5388	0.047	−11.344	0.000	−0.632	−0.445

## 3 Conclusion

Traditional statistical methods remain widely used in clinical trials due to their simplicity and established regulatory acceptance. Estimates of treatment effectiveness obtained using such simple parameter-free models, however, may fail to account for patient variability, in which case they may be biased when applied to out-of-sample groups of patients or individual patients. Analysis of correlation between the primary endpoint’s variation and the patient variability can be performed using multivariable models and employing AI and, in particular, machine learning (ML) modeling. This approach is still mostly limited to personalized medicine, but in fact, it can be easily applied to data analysis in traditional clinical trials. Such synthesis of the personalized medicine methodology with the mass medicine clinical data may provide tangible benefits at virtually no cost: (i) unexplained variation (1 – R^2^) of the primary endpoint can be reduced; (ii) the effect size can be estimated with a smaller RMSE and narrower CI; (iii) predictions of patient outcome could be made more accurately for individual new patients.

Currently, there are extensive FDA documentation and recommendations (Guidance for Industry, 1998; Howell, 2013) related to the utilization of traditional statistical models in clinical trials. ML modeling, though it is in fact an extension and development of the classical statistical methods via more advanced mathematical means, are not regulated by FDA as clearly, though recent publication (U.S. Food and Drug Administration, 2025) made a considerable step in the right direction. In particular, subgroup analysis, which is central to many published clinical trials and thoroughly discussed in FDA documents, may be replaced or complemented by regression analysis or another method of analysis of continuous variables in ML modeling. While FDA documentation mentions possible presence of covariates that correlate with the primary endpoint, insufficient regulatory guidance may impede the FDA approval of clinical trial results that incorporate machine learning in data analysis.

To facilitate the integration of personalized medicine into regulatory practice, the FDA could expand its guidance to include multivariable modeling and AI-based analysis in clinical trials. This includes standardizing the use of ML tools for individualized outcome prediction, requiring reporting on covariate-outcome relationships, and allowing hybrid trial models that incorporate both traditional and AI-driven analyses. Additionally, the FDA should establish performance and validation standards for predictive models, encourage the use of real-world and historical data for training, and support adaptive trial designs guided by AI. Clarifying regulatory definitions for terms like “personalized” and “precision” medicine would further promote consistency and transparency. These steps would ensure a more organized, equitable, and scientifically robust regulatory pathway for personalized medicine. Expanding the guidelines of FDA, EMA, and similar agencies to include ML-based trial designs in a more detailed and explicit fashion would facilitate the integration of personalized medicine into clinical practice, improving treatment efficiency and patient outcomes.
